# Increased disease severity during COVID-19 related hospitalization in black non-hispanic, hispanic and medicaid-insured young children

**DOI:** 10.3389/fped.2024.1373444

**Published:** 2024-06-12

**Authors:** Monica Oyidu Ochapa, Leah J. McGrath, Tamuno Alfred, Santiago M. C. Lopez, Rajeev M. Nepal

**Affiliations:** ^1^ Morgan State University School of Community Health and Policy, Baltimore, MD, United States; ^2^ US Scientific and Medical Affairs, Pfizer Inc., New York, NY, United States; ^3^ Global Medical and Scientific Affairs, Pfizer Inc., New York, NY, United States; ^4^ Statistical Research and Data Science Center, Pfizer Inc., New York, NY, United States

**Keywords:** pediatric COVID-19 severity, health disparities, race/ethnicity, social determinants of health, payer status

## Abstract

**Background:**

The COVID-19 pandemic has disproportionately affected marginalized groups in the United States. Although most children have mild or asymptomatic COVID-19, some experience severe disease and long-term complications. However, few studies have examined health disparities in severe COVID-19 outcomes among US children.

**Objective:**

To examine disparities in the clinical outcomes of infants and children aged <5 years hospitalized with COVID-19 by race/ethnicity and payer status.

**Methods:**

Children aged <5 years hospitalized with an admission diagnosis of COVID-19 (April 2021-February 2023) were selected from the PINC AI™ Healthcare Database. Hospital outcomes included length of stay (LOS), intensive care unit (ICU) admission, oxygen supplementation, invasive mechanical ventilation (IMV), and prolonged duration of each outcome. Multivariable logistic regression models compared hospitalization outcomes by race/ethnicity and payer status.

**Results:**

Among 10,190 children (mean age: 0.9 years, 56.5% male, 66.7% Medicaid-insured), race/ethnicity was distributed as follows: White non-Hispanic (35.1%), Hispanic (any or Unknown race; 28.3%), Black non-Hispanic (15.2%), Other race/ethnicity (8.9%) and Unknown (12.5%). Payer status varied by race/ethnicity. White non-Hispanic children had the highest proportion with commercial insurance (42.9%) while other racial/ethnic groups ranged between 13.8% to 26.1%. Black non-Hispanic children had the highest proportion with Medicaid (82.3%) followed by Hispanic children (76.9%). Black non-Hispanic children had higher odds of prolonged outcomes: LOS (adjusted odds ratio [aOR] = 1.20, 95% confidence interval [CI]:1.05–1.38), ICU days (aOR = 1.44, 95% CI: 1.07–1.93), and IMV days (aOR = 1.80, 95% CI: 1.09–2.97) compared to White non-Hispanic children. Similar patterns were observed for Hispanic and children of Other race/ethnicity. Medicaid-insured and children with other insurance had higher odds of prolonged LOS and oxygen days than commercially insured patients.

**Conclusion:**

There were disparities in clinical outcomes of COVID-19 by race/ethnicity and insurance type, particularly for prolonged-duration outcomes. Further research is required to fully comprehend the causes and consequences of these disparities and develop strategies to reduce them while ensuring equitable healthcare delivery.

## Introduction

A highly transmissible and pathogenic coronavirus called severe acute respiratory syndrome coronavirus 2 (SARS-CoV-2) has caused a pandemic of COVID-19 which continues to cause hospitalization and death (1–3). Racial and ethnic minority groups and lower-income populations have been disproportionately affected by the pandemic (4, 5). The COVID-19 pandemic has exacerbated health inequalities in the United States which were identified early in the pandemic, as lower-income populations had limited access to SARS-CoV-2 testing presumably done by polymerase chain reaction (PCR) diagnostic technique using conventional nasopharyngeal swabs, and once tested were more likely to test positive (6), and rates for COVID-19 hospitalizations and deaths were higher in racial/ethnic minority groups and low-income neighborhoods (7, 8). According to a Centers for Disease Control and Prevention (CDC) report, which examined demographic trends among US COVID-19 mortality from 01 May–31 August 2020, Black and Hispanic persons were disproportionately affected (9). These disparities have persisted, as evident by the cumulative age-adjusted data, which reveals that Black, Hispanic, American Indian, and Alaska Native (AI/AN) persons are more likely than White people to have COVID-19 cases, hospitalizations, and deaths (10). Racial, ethnic, and socioeconomic disparities have been reported in COVID-19 vaccination coverage as well (11); however, some of these disparities have narrowed over time, likely due to vaccination initiatives to provide equitable access to COVID-19 vaccines (12). In addition, insurance status, a proxy for socioeconomic status (13), is also associated with disproportionate burden of COVID-19 illness and death (14). These health disparities reflect the structural and systemic inequities that shape the distribution of health risks and outcomes in the United States.

Studies characterizing inequalities in COVID-19 outcomes in the US have largely focused on the adult populations and there is a paucity of data in pediatric populations. The objective of this study is to examine disparities in clinical outcomes of infants and children under the age of five hospitalized with COVID-19 by race/ethnicity and insurance status.

## Methods

### Study design and data source

This study used a retrospective cohort design to characterize hospitalization outcomes among children aged <5 years and hospitalized with COVID-19 (15). The data source was the nationally representative PINC AI™ (formerly Premier) Healthcare Database Special Edition (PHD SR), which covers approximately a quarter of all hospital admissions annually arising from ∼1,400 hospitals in the U.S. The database contains deidentified administrative healthcare data including admission and discharge diagnoses, hospital characteristics, costs of care, laboratory tests performed and discharge status that are derived from hospital discharge information (16). The secondary data used in this study adhered to the Declaration of Helsinki's ethical principles, and was exempted from Institutional Review Board review under the U.S. Department of Health and Human Services' Policy for Protection of Human Research Subjects category 4 exemption (Sterling IRB, Atlanta, GA).

### Study population

The study population included infants and children aged <5 years hospitalized with a COVID-19 diagnosis (ICD-10-CM U07.1) in any position between April 2021 and February 2023. The COVID-19 diagnosis was required to be “Present on Admission” to limit nosocomial and incidental COVID-19 infections. Other exclusion criteria were (1) Unknown biological sex and (2) newborns with a birth code (Z38.XX) during admission to limit SARS-CoV-2 infection acquired at time of delivery. Only the first qualifying hospital admission during the study period was included (i.e., no readmissions).

### Socio-demographic and clinical characteristics

Demographic and clinical characteristics, including age, sex, race, ethnicity, payer status, and comorbid conditions were assessed. Due to the data structure, age was ascertained in yearly increments; monthly ages of infants aged <1 year were unavailable. The Office of Management and Budget (OMB) race/ethnicity classification was used to categorize the patients by race/ethnicity (17, 18). Race/ethnicity was categorized as a combined variable: Hispanic (Hispanic with any race or Unknown race), White non-Hispanic, Black non-Hispanic, Other (non-Hispanic persons reported as “other” or multiple races or Asian Non-Hispanic), and Unknown race or ethnicity (except known Hispanic with Unknown race) (18). Payer status was categorized into four groups: Commercial, Medicaid, Other (including Medicare due to low numbers of Medicare patients in this age group), and Uninsured. The presence of comorbid conditions was determined using the relevant ICD-10-CM codes in the primary or secondary diagnosis fields.

### Outcomes

Disease severity was based on the outcomes of interest which included length of stay (LOS), intensive care unit (ICU) admission, oxygen supplementation, invasive mechanical ventilatory (IMV) support, and prolonged duration of each outcome. Prolonged duration was defined as longer than the median value among all patients who experienced that outcome (>2 days for each outcome) (15).

### Statistical analyses

Descriptive statistics were used to summarize the demographic characteristics, comorbid conditions, and hospitalization outcomes for each cohort and stratification. Categorical variables were reported as counts and percentages, whereas continuous variables were summarized using means, medians, standard deviations (SD), and interquartile range (IQR). The associations between the exposures of interest (race/ethnicity and payer status) on each COVID-19 outcome were investigated using multivariable logistic regression additionally adjusting for sociodemographic factors, comorbidities, month of admission and hospital location. Unadjusted and adjusted odds ratios (aOR) and 95% confidence intervals (CIs) for each outcome by race/ethnicity and payer status were reported. All statistical analyses were performed using SAS 9.4 (SAS Institute; Cary, NC, USA).

## Results

### Study population

**Figure 1:**
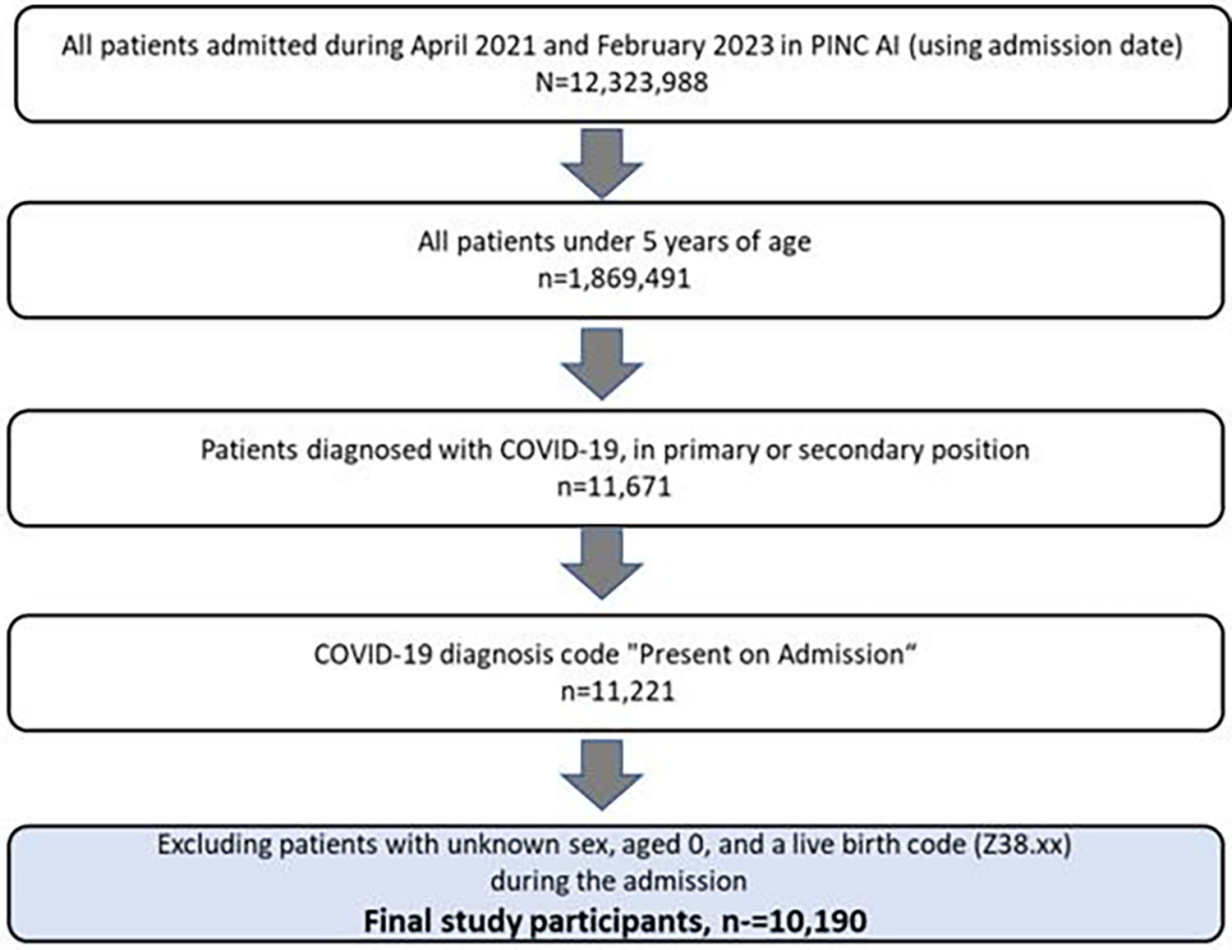
Flow of selection of study cohort.

The patient selection process is illustrated in Figure 1. Out of all the inpatient admissions recorded in the PINC AI between April 2021 and February 2023, 1,869,491 (15.2%) were children aged <5 years, and 11,221 (0.09%) had a COVID-19 diagnosis in the primary or secondary position present on admission. There were 10,190 patients remaining after study exclusion criteria were applied.

### Social-Demographic and clinical characteristics

The demographic and clinical characteristics of the study population by race/ethnicity and payer status is shown in Table 1. The mean age of the cohort was 0.9 year with 56.4% of the children being under the age of one. White non-Hispanic (35.1%) was the most common race/ethnicity among the children, followed by Hispanic (28.3%) and Black non-Hispanic (15.2%); race/ethnicity was Unknown or Other in 12.5% and 8.9% of children, respectively. Most of the children were male (56.5%), had the primary COVID-19 diagnosis (66.6%), and admitted to urban hospitals (95.1%). Medicaid (66.7%) was the most common payer type among hospitalized children followed by commercial (26.6%), Other (5%), and uninsured (1.7%). Payer status varied by race/ethnicity. The proportion of commercially insured children was the highest among White non-Hispanic group (42.9%), while other racial/ethnic groups ranged between 13.8%–26.1%. On the contrary, the proportion of Medicaid insurance was highest among Black non-Hispanic (82.3%) followed by Hispanic (76.9%) and Non-Hispanic White (50.5%). Less than 3% of children were uninsured in all groups.

**Table 1:** Socio-demographic and clinical characteristics of children (<5 years of age) hospitalized with COVID-19.

Characteristics		Race/Ethnicity					Payer status			
	Overall	White non-Hispanic	Hispanic (Hispanic with any or unknown race)	Black, non-Hispanic	Unknown	Other	Commercial	Medicaid	Other	Uninsured
	*N *= 10,190	*N *= 3,573	*N *= 2,884	*N *= 1,551	*N *= 1,271	*N *= 911	*N *= 2,707	*N *= 6,801	*N *= 507	*N *= 175
Age in years										
Mean (SD)	0.9 (1.2)	0.9 (1.3)	0.8 (1.2)	1.0 (1.3)	0.8 (1.2)	1.0 (1.3)	1.0 (1.3)	0.9 (1.2)	0.9 (1.2)	0.8 (1.2)
Median (Q1, Q3)	0 (0, 1)	0 (0, 1)	0 (0, 1)	0 (0, 2)	0 (0, 1)	1 (0, 2)	0 (0, 2)	0 (0, 1)	0 (0, 1)	0 (0, 1)
Age group in years, *n* (%)										
0	5,745 (56.4)	2,020 (56.5)	1,674 (58.0)	826 (53.3)	787 (61.9)	438 (48.1)	1,440 (53.2)	3,939 (57.9)	266 (52.5)	100 (57.1)
1	1,930 (18.9)	665 (18.6)	524 (18.2)	300 (19.3)	229 (18.0)	212 (23.3)	536 (19.8)	1,243 (18.3)	115 (22.7)	36 (20.6)
2	1,066 (10.5)	346 (9.7)	312 (10.8)	193 (12.4)	102 (8.0)	113 (12.4)	307 (11.3)	686 (10.1)	53 (10.5)	20 (11.4)
3	802 (7.9)	308 (8.6)	204 (7.1)	116 (7.5)	87 (6.8)	87 (9.5)	238 (8.8)	512 (7.5)	43 (8.5)	9 (5.1)
4	647 (6.3)	234 (6.5)	170 (5.9)	116 (7.5)	66 (5.2)	61 (6.7)	186 (6.9)	421 (6.2)	30 (5.9)	10 (5.7)
Sex, *n* (%)										
Female	4,436 (43.5)	1,612 (45.1)	1,230 (42.6)	672 (43.3)	527 (41.5)	395 (43.4)	1,150 (42.5)	2,997 (44.1)	221 (43.6)	68 (38.9)
Male	5,754 (56.5)	1,961 (54.9)	1,654 (57.4)	879 (56.7)	744 (58.5)	516 (56.6)	1,557 (57.5)	3,804 (55.9)	286 (56.4)	107 (61.1)
Race/ ethnicity, *n* (%)										
Asian Hispanic	16 (0.2)	0	16 (0.6)	0	0	0	3 (0.1)	12 (0.2)	1 (0.2)	0 (0.0)
Asian Non-Hispanic	311 (3.1)	0	0	0	0	311 (34.1)	111 (4.1)	176 (2.6)	20 (3.9)	4 (2.3)
Black Hispanic	86 (0.8)	0	86 (3.0)	0	0	0	15 (0.6)	65 (1.0)	5 (1.0)	1 (0.6)
Black Non-Hispanic	1,551 (15.2)	0	0	1,551 (100.0)	0	0	214 (7.9)	1,277 (18.8)	40 (7.9)	20 (11.4)
Other Hispanic	987 (9.7)	0	987 (34.2)	0	0	0	138 (5.1)	799 (11.7)	29 (5.7)	21 (12.0)
Other Non-Hispanic	600 (5.9)	0	0	0	0	600 (65.9)	127 (4.7)	436 (6.4)	26 (5.1)	11 (6.3)
White Hispanic	1,578 (15.5)	0	1,578 (54.7)	0	0	0	251 (9.3)	1,161 (17.1)	143 (28.2)	23 (13.1)
White Non-Hispanic	3,573 (35.1)	3,573 (100.0)	0	0	0	0	1,534 (56.7)	1,804 (26.5)	180 (35.5)	55 (31.4)
Unknown^a^	1,488 (14.6)	0	217 (7.5)	0	1,271 (100.0)	0	314 (11.6)	1,071 (15.7)	63 (12.4)	40 (22.9)
Insurance type, *n* (%)										
Commercial	2,707 (26.6)	1,534 (42.9)	435 (15.1)	214 (13.8)	286 (22.5)	238 (26.1)	2,707 (100.0)	0	0	0
Medicaid	6,801 (66.7)	1,804 (50.5)	2,217 (76.9)	1,277 (82.3)	891 (70.1)	612 (67.2)	0	6,801 (100.0)	0	0
Other^b^	507 (5.0)	180 (5.0)	182 (6.3)	40 (2.6)	59 (4.6)	46 (5.0)	0	0	507 (100.0)	0
Uninsured	175 (1.7)	55 (1.5)	50 (1.7)	20 (1.3)	35 (2.8)	15 (1.6)	0	0	0	175 (100.0)
Hospital urbanicity, *n* (%)										
Rural	501 (4.9)	229 (6.4)	96 (3.3)	76 (4.9)	48 (3.8)	52 (5.7)	138 (5.1)	315 (4.6)	34 (6.7)	14 (8.0)
Urban	9,689 (95.1)	3,344 (93.6)	2,788 (96.7)	1,475 (95.1)	1,223 (96.2)	859 (94.3)	2,569 (94.9)	6,486 (95.4)	473 (93.3)	161 (92.0)
Diagnosis position, *n* (%)										
Primary	6,789 (66.6)	2,387 (66.8)	1,935 (67.1)	1,006 (64.9)	863 (67.9)	598 (65.6)	1,805 (66.7)	4,508 (66.3)	366 (72.2)	110 (62.9)
Secondary	3,401 (33.4)	1,186 (33.2)	949 (32.9)	545 (35.1)	408 (32.1)	313 (34.4)	902 (33.3)	2,293 (33.7)	141 (27.8)	65 (37.1)
Comorbid conditions, *n* (%)										
Diabetes	61 (0.6)	33 (0.9)	10 (0.3)	6 (0.4)	5 (0.4)	7 (0.8)	28 (1.0)	29 (0.4)	2 (0.4)	2 (1.1)
Obesity/ overweight	50 (0.5)	12 (0.3)	25 (0.9)	5 (0.3)	3 (0.2)	5 (0.5)	5 (0.2)	42 (0.6)	3 (0.6)	0
Hypertension	142 (1.4)	30 (0.8)	43 (1.5)	31 (2.0)	19 (1.5)	19 (2.1)	23 (0.8)	117 (1.7)	1 (0.2)	1 (0.6)
Neurological disease	660 (6.5)	214 (6.0)	192 (6.7)	113 (7.3)	76 (6.0)	65 (7.1)	148 (5.5)	483 (7.1)	26 (5.1)	3 (1.7)
Asthma/reactive airway	801 (7.9)	279 (7.8)	181 (6.3)	184 (11.9)	87 (6.8)	70 (7.7)	195 (7.2)	569 (8.4)	30 (5.9)	7 (4.0)
Down syndrome/ chromosomal anomaly	153 (1.5)	58 (1.6)	51 (1.8)	13 (0.8)	17 (1.3)	14 (1.5)	44 (1.6)	103 (1.5)	6 (1.2)	0
Immunocompromised	1,573 (15.4)	463 (13.0)	426 (14.8)	331 (21.3)	200 (15.7)	153 (16.8)	400 (14.8)	1,087 (16.0)	72 (14.2)	14 (8.0)
Solid malignancy	177 (1.7)	72 (2.0)	52 (1.8)	21 (1.4)	19 (1.5)	13 (1.4)	50 (1.8)	117 (1.7)	9 (1.8)	1 (0.6)
Hematologic malignancy	109 (1.1)	43 (1.2)	36 (1.2)	8 (0.5)	9 (0.7)	13 (1.4)	36 (1.3)	68 (1.0)	5 (1.0)	0
Rheumatologic/other inflammatory condition	348 (3.4)	96 (2.7)	114 (4.0)	54 (3.5)	49 (3.9)	35 (3.8)	97 (3.6)	216 (3.2)	29 (5.7)	6 (3.4)
Primary immunodeficiency	342 (3.4)	118 (3.3)	92 (3.2)	58 (3.7)	35 (2.8)	39 (4.3)	86 (3.2)	241 (3.5)	13 (2.6)	2 (1.1)
Chronic kidney or end-stage renal disease	299 (2.9)	91 (2.5)	80 (2.8)	53 (3.4)	45 (3.5)	30 (3.3)	63 (2.3)	219 (3.2)	14 (2.8)	3 (1.7)
Other immune condition	688 (6.8)	187 (5.2)	159 (5.5)	202 (13.0)	81 (6.4)	59 (6.5)	184 (6.8)	477 (7.0)	24 (4.7)	3 (1.7)
Metabolic disease	91 (0.9)	43 (1.2)	20 (0.7)	11 (0.7)	9 (0.7)	8 (0.9)	17 (0.6)	65 (1.0)	9 (1.8)	0
Sickle cell	191 (1.9)	4 (0.1)	19 (0.7)	128 (8.3)	32 (2.5)	8 (0.9)	34 (1.3)	151 (2.2)	6 (1.2)	0
Psychiatric disorders	15 (0.1)	5 (0.1)	4 (0.1)	1 (0.1)	4 (0.3)	1 (0.1)	2 (0.1)	13 (0.2)	0	0
Congenital heart condition	42 (0.4)	13 (0.4)	15 (0.5)	6 (0.4)	3 (0.2)	5 (0.5)	13 (0.5)	27 (0.4)	2 (0.4)	0
Congenital lung condition	29 (0.3)	8 (0.2)	11 (0.4)	7 (0.5)	1 (0.1)	2 (0.2)	5 (0.2)	22 (0.3)	2 (0.4)	0
Autoimmune disease	65 (0.6)	34 (1.0)	10 (0.3)	7 (0.5)	5 (0.4)	9 (1.0)	29 (1.1)	32 (0.5)	2 (0.4)	2 (1.1)
Transplant (Bone marrow/organ)	48 (0.5)	11 (0.3)	23 (0.8)	7 (0.5)	2 (0.2)	5 (0.5)	6 (0.2)	39 (0.6)	3 (0.6)	0
Disability^c^	88 (0.9)	40 (1.1)	27 (0.9)	11 (0.7)	4 (0.3)	6 (0.7)	20 (0.7)	63 (0.9)	3 (0.6)	2 (1.1)

^a^ Unknown refers to either one, or both race and ethnicity are unknown.

^b^ Includes Medicare.

^c^ Neurologic, neurodevelopmental, intellectual, physical, vision or hearing impairment.

Q1, 25th percentile; Q3, 75th percentile; SD, standard deviation.

Other characteristics included in the models were calendar month of admission and hospital location.

As shown on Table 1, the most common comorbid conditions were immunocompromising status (15.4%), asthma/reactive airway (7.9%), and neurological disease (6.5%). The most common immunocompromising conditions were rheumatologic/other inflammatory condition (3.4%), primary immune-deficiency (3.4%), chronic kidney or end-stage renal disease (2.9%), solid malignancy (1.7%), hematologic malignancy (1.1%), and other condition (6.8%). The prevalence of comorbid conditions varied among races/ethnicities. Some comorbid conditions were more prevalent among Black non-Hispanic than other races/ethnicities. Comorbid conditions which had the highest prevalence among Black non-Hispanic and the lowest among White non-Hispanic were immunocompromising condition (21.3% vs. 13%), neurological disease (7.3% vs. 6%), and sickle cell (8.3% vs. 0.1%). The prevalence of asthma/reactive airway ranged was 11.9% among Black non-Hispanic children and 6.3% among Hispanic children. Rheumatologic/ other inflammatory condition (4%) was the most common among Hispanic children. Comorbid conditions were infrequent among White non-Hispanic children with solid malignancy being the most prevalent (2%). The prevalence of comorbid conditions also varied by payer status. Notably, some conditions were most prevalent among Medicaid and least prevalent among uninsured which included immunocompromising status (16% vs. 8%) and asthma/reactive airway (8.4% vs. 4%).

### Clinical outcomes

**Figure 2:**
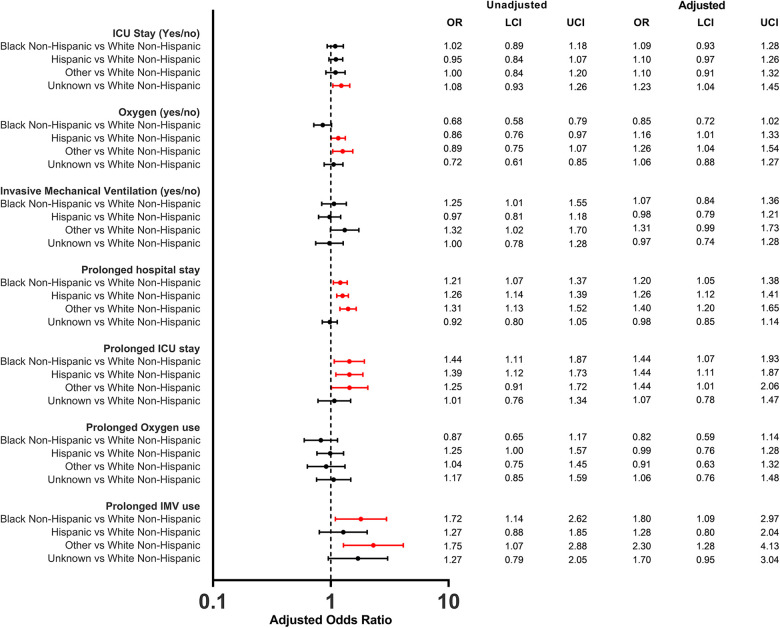
Odds ratios of in-hospital outcomes among children (<5 years of age) hospitalized with COVID-19 by race/ethnicity. The reference group for all models were White Non-Hispanic children.

**Figure 3:**
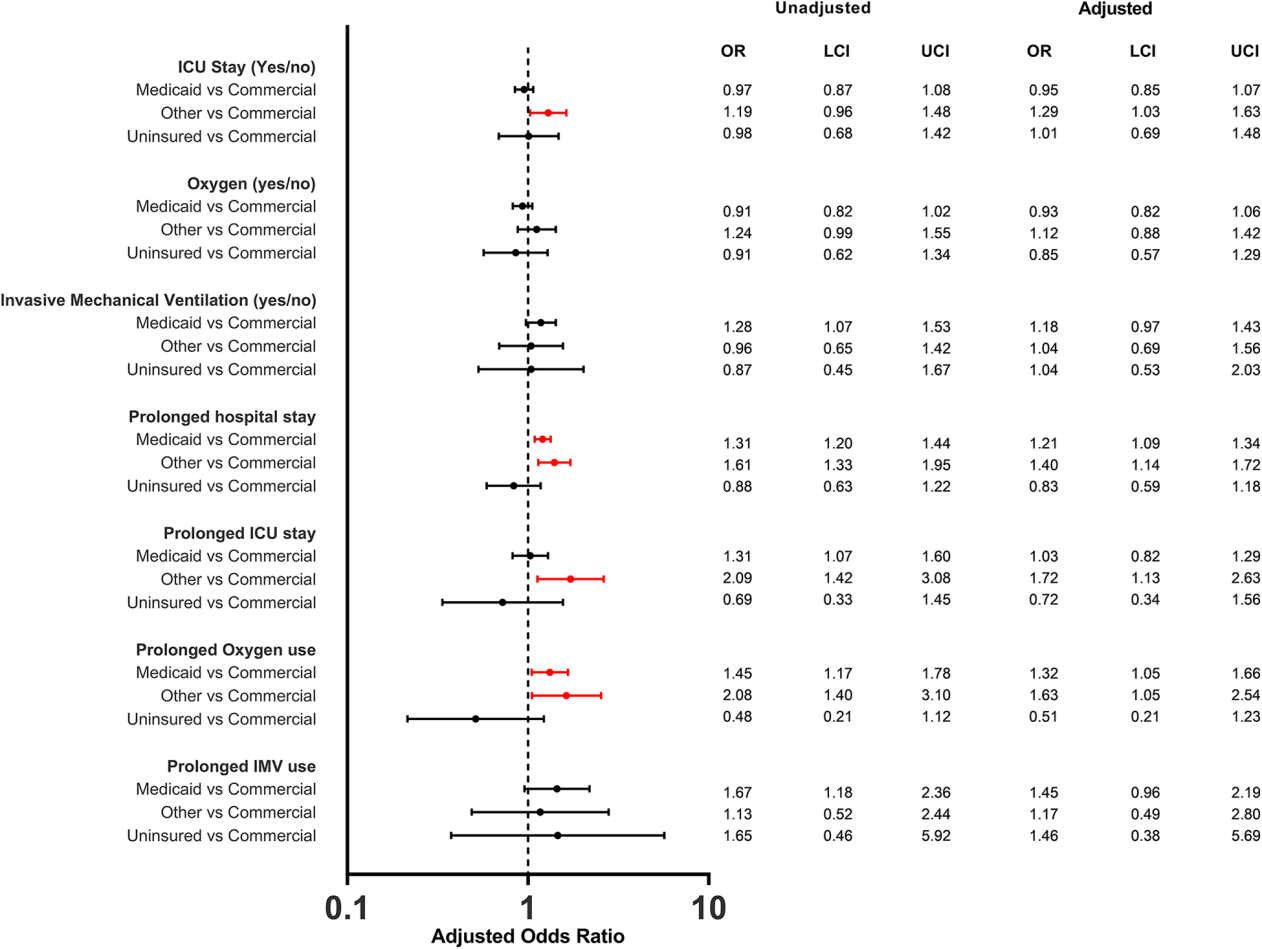
Odd ratios of in-hospital outcomes among children (<5 years of age) hospitalized with COVID-19 by payer status.

The distribution of clinical outcomes, overall and stratified by race/ethnicity and payer status are shown in Table 2. The overall median length of hospital stay for children was 2 days (IQR, 1–3), and was consistent across different races/ethnicities and payer types although IQR slightly varied. Overall, 37.9% of children had prolonged length of hospital stay. Adjusted logistic regression analysis showed that Black non-Hispanic children (aOR: 1.2; 95% CI 1.1–1.4), Hispanic children (aOR: 1.3; 95% CI 1.1–1.4) and children of other races (aOR: 1.4; 95% CI 1.2–1.6) were more likely to have a prolonged length of stay compared to White non-Hispanic children (Figure 2). When analyzed by payer type, children with Medicaid (aOR: 1.2; 95% CI 1.1–1.3) and other insurances (aOR: 1.4; 95% CI 1.1–1.7) were more likely to have prolonged length of stay compared to children with commercial insurance (Figure 3).

**Table 2:** Clinical outcomes among children (<5 years of age) hospitalized with COVID-19.

Characteristics		Race/Ethnicity					Payer status			
	Overall	White non-Hispanic	Hispanic (Hispanic with any or unknown race)	Black, non-Hispanic	Unknown	Other	Commercial	Medicaid	Other	Uninsured
	*N *= 10,190	*N *= 3,573	*N *= 2,884	*N *= 1,551	*N *= 1,271	*N *= 911	*N *= 2,707	*N *= 6,801	*N *= 507	*N *= 175
Length of stay in days										
Mean (SD)	3.4 (6.3)	3.1 (5.2)	3.4 (4.8)	3.9 (8.2)	3.5 (9.1)	3.7 (5.9)	3.0 (4.7)	3.6 (7.0)	3.5 (4.5)	2.9 (4.1)
Median (Q1, Q3)	2 (1, 3)	2 (1, 3)	2 (1, 4)	2 (1, 4)	2 (1, 3)	2 (1, 4)	2 (1, 3)	2 (1, 4)	2 (1, 4)	2 (1, 3)
Prolonged length of stay, *n* (%)	3,858 (37.9)	1,262 (35.3)	1,175 (40.7)	617 (39.8)	424 (33.4)	380 (41.7)	897 (33.1)	2,683 (39.5)	225 (44.4)	53 (30.3)
ICU admission, *n* (%)	2,230 (21.9)	782 (21.9)	607 (21.0)	346 (22.3)	295 (23.2)	200 (22.0)	598 (22.1)	1,466 (21.6)	128 (25.2)	38 (21.7)
Length of ICU stay in days										
*n* ^a^	2,204	774	600	344	288	198	589	1,450	127	38
Mean (SD)	4.1 (8.5)	3.6 (6.8)	4.0 (5.6)	5.3 (13.0)	4.7 (12.0)	3.9 (5.6)	3.5 (6.6)	4.5 (9.5)	3.5 (4.0)	3.1 (4.5)
Median (Q1, Q3)	2 (1, 4)	2 (1, 3)	2 (1, 4)	2 (1, 4)	2 (1, 3.5)	2 (1, 4)	2 (1, 3)	2 (1, 4)	3 (1, 4)	1 (1, 3)
Prolonged ICU stay, *n* (%)	863 (39.2)	272 (35.1)	258 (43.0)	151 (43.9)	102 (35.4)	80 (40.4)	201 (34.1)	586 (40.4)	66 (52.0)	10 (26.3)
Supplemental oxygen use, *n* (%)	2,048 (20.1)	806 (22.6)	578 (20.0)	255 (16.4)	221 (17.4)	188 (20.6)	566 (20.9)	1,323 (19.5)	125 (24.7)	34 (19.4)
Length of supplemental oxygen in days										
*n* ^a^	1,932	761	541	243	206	181	539	1,238	121	34
Mean (SD)	3.1 (4.4)	3.0 (4.1)	3.3 (3.9)	2.8 (2.9)	3.7 (6.7)	3.4 (5.0)	2.8 (3.1)	3.3 (4.9)	3.5 (3.2)	2.6 (2.8)
Median (Q1, Q3)	2 (1, 4)	2 (1, 3)	2 (1, 4)	2 (1, 3)	2 (2, 4)	2 (1, 4)	2 (1, 3)	2 (1, 4)	3 (2, 4)	2 (1, 2)
Prolonged supplemental oxygen, *n* (%)	803 (41.6)	304 (39.9)	246 (45.5)	89 (36.6)	90 (43.7)	74 (40.9)	189 (35.1)	543 (43.9)	64 (52.9)	7 (20.6)
IMV, *n* (%)	778 (7.6)	259 (7.2)	204 (7.1)	138 (8.9)	92 (7.2)	85 (9.3)	177 (6.5)	559 (8.2)	32 (6.3)	10 (5.7)
Length of IMV in days										
*n* ^a^	772	257	201	138	92	84	175	555	32	10
Mean (SD)	5.3 (9.2)	4.3 (6.1)	5.2 (8.5)	6.4 (10.7)	7.2 (14.9)	4.6 (6.8)	3.8 (5.0)	5.8 (10.3)	3.6 (4.2)	4.9 (6.7)
Median (Q1, Q3)	2 (1, 5)	2 (1, 5)	2 (1, 5)	3 (1, 6)	2 (1, 5)	3 (1, 5)	2 (1, 4)	3 (1, 6)	2 (1, 4)	2.5 (2, 4)
Prolonged IMV, *n* (%)	363 (47.0)	105 (40.9)	94 (46.8)	75 (54.3)	43 (46.7)	46 (54.8)	66 (37.7)	279 (50.3)	13 (40.6)	5 (50.0)

^a^ Number of patients with the outcome and the number of service days available.

ICU, intensive care unit; IMV, invasive mechanical ventilation; Q1, 25th percentile; Q3, 75th percentile; SD, standard deviation.

There were 2,230 (21.9%) children admitted to the ICU. The proportion of ICU admission only slightly varied by race/ethnicity and payer status (Table 1). The overall median length of ICU stay was 2 days (IQR, 1–4) and was consistent across races/ethnicities although IQR slightly varied. By payer type, median length of ICU stay was 2 days (IQR, 1–3) for commercial, 2 days (IQR, 1–4) for Medicaid, 1 day (IQR, 1–3) for non-insured, and 3 days (IQR, 1–4) for Other. The proportion of children with prolonged ICU stay was 39.2%. Black non-Hispanic (aOR: 1.4; 95% CI 1.1–1.9), Hispanic (aOR: 1.4; 95% CI 1.1–1.9) and other races (aOR: 1.4; 95% CI 1.0–2.1) had higher odds of having a prolonged length of ICU stay compared to White non-Hispanic (Figure 2). When analyzed by payer type, only those with other insurance had higher odds (aOR: 1.7; 95% CI 1.1–2.6) of having a prolonged length of ICU stay compared to commercial (Figure 3).

The overall proportion of infants and children that required IMV during COVID-19 hospitalization was 7.6% (*n *= 778). The proportion varied among races/ethnicities and ranged between 9.3% (Other) and 7.1% (Hispanic). By payer type, it ranged between 8.2% (Medicaid) and 5.7% (non-insured). The overall median length of IMV use was 2 days (IQR, 1–5) and consistent among races/ethnicities, except for Non-Hispanic blacks (median 3 days, IQR: 1–6) and other races (median 3 days, IQR: 1–5). By payer status also the median length of IMV use ranged between 2 and 3 days. Black non-Hispanic children (aOR: 1.8; 95% CI 1.1–3.0), and children from Other races (aOR: 2.3; 95% CI 1.3–4.1) were more likely to have prolonged IMV use compared to White non-Hispanic children while there was no evidence for differences by payer status.

Supplemental oxygen was used by 2,048 (20.1%) of infants and children hospitalized with COVID-19. The proportion of supplemental oxygen use varied both by race/ethnicity (ranged between White non-Hispanic 22.6% and Black non-Hispanic 17.7%) and by payer type (ranged between Unknown 24.7% and uninsured 19.4%). However, after adjustment, Hispanic children (aOR: 1.2; 95% CI 1.0–1.3) and children from Other races (aOR: 1.3; 95% CI 1.0–1.5) had higher odds of supplemental oxygen use compared to White non-Hispanic children. The overall median length of supplemental oxygen use was 2 days (IQR, 1–4). Medicaid insured (aOR: 1.3; 95% CI 1.0–1.7) and other insurance groups (aOR: 1.6; 95% CI 1.1–2.5) had higher odds of prolonged use of supplemental oxygen, however no evidence for differences was observed among races/ethnicities.

## Discussion

This retrospective study examined the disparities in clinical outcomes of a nationally representative cohort of infants and children (*n *= 10,190) aged under 5 years hospitalized with COVID-19 by race/ethnicity and insurance type. Results of this study indicate that Black non-Hispanic, Hispanic, and Other race/ethnicities had higher odds of prolonged-hospital and -ICU stay than White non-Hispanic children. Similarly, children with Medicaid and Other insurance, independent of their race/ethnicity, had higher odds of having a prolonged hospital stay than children with commercial insurance. In addition, we also observed disparities in the use of oxygen and IMV by race/ethnicity and insurance type. These results suggest that there are differences in the clinical outcomes of children hospitalized with COVID-19 by race/ethnicity and insurance type, mirroring the trends observed among the adult populations (4).

In our analysis, we have used length of hospital stay, ICU stay and the use of IMV and supplemental oxygen as a representation of disease severity for COVID-19. The overall median length of hospital stay, and ICU stay for children was 2 days for each outcome and consistent across races/ethnicities. We modelled the dichotomous measure of duration (prolonged vs. non prolonged). Adjusted statistical analysis showed that Black non-Hispanic children, Hispanic children and children of other races had higher odds of prolonged length of both hospital stay and ICU stay compared to White non-Hispanic children. We saw more variability in median length of IMV days across study groups ranging from 2 days for White non-Hispanic, Hispanic, and other races to 3 days for Black non-Hispanic, and unknown races. Upon statistical analysis, prolonged IMV days showed similar association as hospital/ICU prolonged stay with the race/ethnicity except for Hispanic children. However, the results are less clear with the use of oxygen. We did not see any association between prolonged oxygen use and race/ethnicity. Interestingly, although it did not meet statistical significance, there was a higher tendency for White non-Hispanic patients to be on oxygen compared to Black non-Hispanic. This could be because of racial and ethnic discrepancy in delayed identification of treatment eligibility among patients with COVID-19 as reported earlier (19).

Our findings are consistent with previous studies that assessed ethnic and racial disparities in pediatric COVID-19 hospitalizations and outcomes (20). A CDC report which included results from March 2020–March 2022 estimated that Black, Hispanic, and American Indian & Alaska Native (AI/AN) children were twice as likely to be hospitalized compared to Non-Hispanic White children (21). Another CDC study among US individuals aged <21 years reported that Hispanic, Black, and AI/AN persons accounted for 75% of COVID-19 deaths in persons aged <21 years although these groups represented only 41% of the US population in this age group (22). A large population-based study from England also showed that Black children and children of mixed or other backgrounds had significantly longer hospital stay compared with White children (23).

Association between insurance status/type and pediatric COVID-19 outcomes is less well defined because of paucity of data. However, our findings are consistent with the results obtained from studies on adult populations. A retrospective cohort analysis of COVID-19 patients at Sutter Health system in northern California reported that compared to individuals with Commercial insurance, those with Medicaid, self-pay patients, or no reported insurance had a twice as high likelihood of admission for COVID-19 (24, 25). Having access to insurance lowered the risk of dying from COVID-19 but had no significant association with the rate of infection (26).

Results from COVID-19 studies have heightened the pre-existing health disparities in the US. These disparities in COVID-19 outcomes among different racial and ethnic communities have not been conclusively attributed to any genetic or epigenetic markers, although it is plausible that such biological factors may impact the COVID-19 outcomes along with the non-biological factors. These are likely the results of underlying social determinants of health that affect the risk of exposure, transmission, and severity of COVID-19 among children from minority communities (27, 28). These factors include persistent poverty, crowded living conditions, limited access to health care, lack of health insurance, language barriers, cultural beliefs, and discrimination (29). Moreover, children from minority communities may have higher rates of comorbidities that increase the risk of severe COVID-19, such as asthma, obesity, diabetes, and immunosuppression (29). These factors reflect the longstanding structural and systemic inequities.

Our study has several limitations. First, we used data from a single database that may only be representative of some pediatric COVID-19 hospitalizations in the US. Second, we did not have information on some critical variables that may affect outcomes, such as SARS-CoV-2 variant, number of household contacts, and any treatments received in an outpatient setting prior to admission. Third, COVID-19 vaccination status for the children or for the mother (which is more relevant for children <1 year) was unavailable. COVID-19 vaccination was first authorized for children 6 months to 5 or 6 years of age group on June 17, 2022. Approximately 35% of children included in this study were recruited between July 2022 and February 2023. Considering that only 5.1% of US children aged 6 months to 5 years had completed the primary series of COVID-19 vaccinations as of 31 December 2022 (30), this would likely not have impacted the severity of endpoints in this study. However, it is important to note that there are disparities in COVID-19 vaccination uptake by race/ethnicity in the US (31) which could affect the risk of infection and hospitalization for different groups of children. Fourth, children may have been admitted for reasons other than COVID-19. Requiring that the COVID-19 diagnosis be present on admission may have limited some incidental infections. Fifth, for some outcomes (such as prolonged IMV) lack of associations and wide 95% CIs may have been due to a low number of events. Sixth, information about race was unknown for 12.5% of the children. In addition, there may be residual confounding as social economic status variables other than payer status were not available. Finally, information from other facilities for children who transferred was not available, therefore the duration of some endpoints may be undercounted.

## Conclusion

This study revealed disparities in the clinical outcomes of infants and children hospitalized with COVID-19 by race/ethnicity, and insurance type. These findings underscore the need for more research to understand the causes and consequences of these disparities and develop strategies to reduce them. Moreover, these findings emphasize the importance of ensuring equitable access to preventive measures, testing, treatment, and vaccination for pediatric COVID-19 patients from vulnerable populations.

## Data Availability

The raw data supporting the conclusions of this article will be made available by the authors, without undue reservation.

## References

[1] Centers for Disease Control and Prevention. Risk for COVID-19 Infection, Hospitalization, and Death by Race/Ethnicity. (2023).

[2] WHO COVID-19 Dashboard. World Health Organization. (2020). (cited September 1, 2023). Available online at: https://covid19.who.int/

[3] COVID Data Tracker. U.S. Department of Health and Human Services, CDC. (2023). (cited September 1, 2023). Available online at: https://covid.cdc.gov/covid-data-tracker

[4] Magesh S John D Li WT Li Y Mattingly-App A Jain S Disparities in COVID-19 outcomes by race, ethnicity, and socioeconomic status: a systematic-review and meta-analysis. JAMA Netw Open. (2021) 4(11):e2134147. 10.1001/jamanetworkopen.2021.34147 34762110 PMC8586903

[5] Khanijahani A Iezadi S Gholipour K Azami-Aghdash S Naghibi D. A systematic review of racial/ethnic and socioeconomic disparities in COVID-19. Int J Equity Health. (2021) 20(1):248. 10.1186/s12939-021-01582-4 34819081 PMC8611382

[6] Lieberman-Cribbin W Tuminello S Flores RM Taioli E. Disparities in COVID-19 testing and positivity in New York city. Am J Prev Med. (2020) 59(3):326–32. 10.1016/j.amepre.2020.06.005 32703702 PMC7316038

[7] Garg S Kim L Whitaker M O'Halloran A Cummings C Holstein R Hospitalization rates and characteristics of patients hospitalized with laboratory-confirmed coronavirus disease 2019—COVID-NET, 14 states, March 1–30, 2020. MMWR Morb Mortal Wkly Rep. (2020) 69(15):458–64. 10.15585/mmwr.mm6915e3 32298251 PMC7755063

[8] Wadhera RK Wadhera P Gaba P Figueroa JF Joynt Maddox KE Yeh RW Variation in COVID-19 hospitalizations and deaths across New York city boroughs. JAMA. (2020) 323(21):2192–5. 10.1001/jama.2020.7197 32347898 PMC7191469

[9] Gold JAW Rossen LM Ahmad FB Sutton P Li Z Salvatore PP Race, ethnicity, and age trends in persons who died from COVID-19—united States, may–august 2020. MMWR Morb Mortal Wkly Rep. (2020) 69(42):1517–21. 10.15585/mmwr.mm6942e1 33090984 PMC7583501

[10] Centers for Disease Control and Prevention. Risk for COVID-19 Infection, Hospitalization, and Death by Race/Ethnicity: Centers for Disease Control and Prevention; (2023). (updated 2023-05-25). Available online at: https://archive.cdc.gov/#/details?url=https://www.cdc.gov/coronavirus/2019-ncov/covid-data/investigations-discovery/hospitalization-death-by-race-ethnicity.html

[11] Cheng Z Li Y. Racial and ethnic and income disparities in COVID-19 vaccination among medicare beneficiaries. J Am Geriatr Soc. (2022) 70(9):2638–45. 10.1111/jgs.17920 35639044 PMC9348368

[12] Kriss JL Hung MC Srivastav A Black CL Lindley MC Lee JT COVID-19 vaccination coverage, by race and ethnicity—national immunization survey adult COVID module, United States, December 2020–November 2021. MMWR Morb Mortal Wkly Rep. (2022) 71(23):757–63. 10.15585/mmwr.mm7123a2 35679179 PMC9181054

[13] Trinidad S Kotagal M. Socioeconomic factors and pediatric injury. Curr Trauma Rep. (2023) 9(2):47–55. 10.1007/s40719-023-00251-x 36714450 PMC9868497

[14] Campbell T Galvani AP Friedman G Fitzpatrick MC. Exacerbation of COVID-19 mortality by the fragmented United States healthcare system: a retrospective observational study. Lancet Reg Health Am. (2022) 12:100264. 10.1016/j.lana.2022.100264 35582265 PMC9098098

[15] McGrath LJ Moran MM Alfred T Reimbaeva M Di Fusco M Khan F Clinical outcomes of COVID-19 and influenza in hospitalized children &lt;5 years in the US. Front Pediatr. (2023) 11:1261046. 10.3389/fped.2023.1261046 37753191 PMC10518399

[16] PINC AI™ Applied Sciences. PINC AI™ Healthcare Database: Data that Informs and Performs (White Paper). Premier Inc; (2023).

[17] Office of Minority H. Explanation of Data Standards for Race, Ethnicity, Sex, Primary Language, and Disability. Department of Health and Human Services.

[18] Van Dyke ME Mendoza MCB Li W Parker EM Belay B Davis EM Racial and ethnic disparities in COVID-19 incidence by age, sex, and period among persons aged <25 years—16 U.S. Jurisdictions, January 1–December 31, 2020. MMWR MorbMortal Wkly Rep. (2021) 70(11):382–8. 10.15585/mmwr.mm7011e1 PMC7976617 33735165

[19] Fawzy A Wu TD Wang K Robinson ML Farha J Bradke A Racial and ethnic discrepancy in pulse oximetry and delayed identification of treatment eligibility among patients with COVID-19. JAMA Intern Med. (2022) 182(7):730–8. 10.1001/jamainternmed.2022.1906 35639368 PMC9257583

[20] Vicetti Miguel CP Dasgupta-Tsinikas S Lamb GS Olarte L Santos RP. Race, ethnicity, and health disparities in US children with COVID-19: a review of the evidence and recommendations for the future. J Pediatric Infect Dis Soc. (2022) 11(Supplement_4):S132–S40. 10.1093/jpids/piac099 36063366 PMC9494369

[21] Oliveira CR Feemster KA Ulloa ER. Pediatric COVID-19 health disparities and vaccine equity. J Pediatric Infect Dis Soc. (2022) 11(Supplement_4):S141–S7. 10.1093/jpids/piac091 36124679 PMC9494479

[22] Bixler D Miller AD Mattison CP Taylor B Komatsu K Peterson Pompa X SARS-CoV-2-Associated deaths among persons aged <21 years—United States, February 12–July 31, 2020. MMWR Morb Mortal Wkly Rep. (2020) 69(37):1324–9. 10.15585/mmwr.mm6937e4 32941417

[23] Saatci D Ranger TA Garriga C Clift AK Zaccardi F Tan PS Association between race and COVID-19 outcomes among 2.6 million children in England. JAMA Pediatr. (2021) 175(9):928–38. 10.1001/jamapediatrics.2021.1685 34152371 PMC8218232

[24] Azar KMJ Shen Z Romanelli RJ Lockhart SH Smits K Robinson S Disparities in outcomes among COVID-19 patients in a large health care system in California. Health Aff (Millwood). (2020) 39(7):1253–62. 10.1377/hlthaff.2020.00598 32437224

[25] Yehia BR Winegar A Fogel R Fakih M Ottenbacher A Jesser C Association of race with mortality among patients hospitalized with coronavirus disease 2019 (COVID-19) at 92 US hospitals. JAMA Netw Open. (2020) 3(8):e2018039. 10.1001/jamanetworkopen.2020.18039 32809033 PMC7435340

[26] Abedi V Olulana O Avula V Chaudhary D Khan A Shahjouei S Racial, economic, and health inequality and COVID-19 infection in the United States. J Racial Ethn Health Disparities. (2021) 8(3):732–42. 10.1007/s40615-020-00833-4 32875535 PMC7462354

[27] White A Liburd LC Coronado F. Addressing racial and ethnic disparities in COVID-19 among school-aged children: are we doing enough? Prev Chronic Dis. (2021) 18:E55. 10.5888/pcd18.210084 34081577 PMC8220967

[28] Kim L Whitaker M O'Halloran A Kambhampati A Chai SJ Reingold A Hospitalization rates and characteristics of children aged <18 years hospitalized with laboratory-confirmed COVID-19—COVID-NET, 14 states, march 1–July 25, 2020. MMWR Morb Mortal Wkly Rep. (2020) 69(32):1081–8. 10.15585/mmwr.mm6932e3 32790664 PMC7440125

[29] Delahoy MJ Ujamaa D Whitaker M O'Halloran A Anglin O Burns E Hospitalizations associated with COVID-19 among children and adolescents—COVID-NET, 14 states, march 1, 2020–August 14, 2021. MMWR Morb Mortal Wkly Rep. (2021) 70(36):1255–60. 10.15585/mmwr.mm7036e2 34499627 PMC8437052

[30] Murthy BP Fast HE Zell E Murthy N Meng L Shaw L COVID-19 vaccination coverage and demographic characteristics of infants and children aged 6 months-4 years—United States, June 20–December 31, 2022. MMWR Morb Mortal Wkly Rep. (2023) 72(7):183–9. 10.15585/mmwr.mm7207a4 36795658 PMC9949848

[31] Nguyen LH Joshi AD Drew DA Merino J Ma W Lo CH Self-reported COVID-19 vaccine hesitancy and uptake among participants from different racial and ethnic groups in the United States and United Kingdom. Nat Commun. (2022) 13(1):636. 10.1038/s41467-022-28200-3 35105869 PMC8807721

